# Cell Specific Expression of Vascular Endothelial Growth Factor Receptor-2 (Flk-1/KDR) in Developing Mice Embryo and Supporting Maternal Uterine Tissue during Early Gestation (D4-D7)

**DOI:** 10.22074/IJFS.2021.134530

**Published:** 2021-03-11

**Authors:** Dimpimoni Das, Purba J Saikia, Upasa Gowala, Hirendra N Sarma

**Affiliations:** 1 Molecular Endocrinology and Reproductive Biology Research Laboratory, Department of Zoology, Rajiv Gandhi University, Itanagar, Arunachal Pradesh, India; 2 Department of Zoology, Dhemaji College, Dhemaji, Assam, India

**Keywords:** Decidualization, Embryo, Flk-1/KDR, Implantation, Vascular Endothelial Growth Factor

## Abstract

**Background:**

Vascular endothelial growth factor (VEGF) and the corresponding receptors play key role in vascu- logenesis and angiogenesis processes. VEGF is one of the prime candidates in regulating embryo implantation by increasing vascular permeability. VEGF receptor-2, also called Flk-1/KDR, is one of the prime receptor which is actively involved in the execution of various functions of VEGF. However, precise role of this receptor during early gestation period is yet to be addressed. In the present study, expression of Flk-1/KDR during peri-implantation mice uterus as well as fetal-maternal tissues from day 4-day 7 (D4-D7) of gestation was investigated.

**Materials and Methods:**

In this experimental study, localization of Flk-1/KDR was investigated by immunohisto- chemistry and immunofluorescence techniques, in paraffin embedded tissue sections. Flk-1/KDR protein and mRNA expressions were investigated by western blotting and quantitative reverse transcription polymerase chain reaction (qRT-PCR), respectively. Effects of ovarian steroids on expression of Flk-1/KDR were also assessed by estrogen and progesterone antagonist treatment.

**Results:**

Uterine tissue on D4 showed strong expression of Flk-1/KDR in luminal and uterine glandular epithelium. On D5 and D6, differential expression of Flk-1/KDR was evidenced in certain cell types of the embryo, maternal tissues and fetal-maternal interface with varied intensity. Flk-1/KDR was specifically expressed in the ectoplacental cone (EPC) and various cells of the embryo on D7. Flk-1/KDR expression was not evidenced in the estradiol-17β (E2) and progesterone (P4) antagonist treated uterus. Western blotting result revealed presence of Flk-1/KDR protein in the all gestation days, except antagonist treated uterus. qRT-PCR analysis showed significant increase of Flk-1/KDR mRNA transcript on D6 and D7.

**Conclusion:**

Spatial-temporal expression of Flk-1/KDR during peri-implntation period in mice uterus especially in the feto-maternal interface was observed. This spatio-temporal specificity as well as increased expression of Flk-1/KDR could be one of the determinants for establishment of fetal-maternal cross talk during the critical period of develop- ment.

## Introduction

A road map of embryo growth, implantation and further development in laboratory model system (rodents) is being a matter of biological investigation* in vivo*, during the last few decades. Roles of ovarian hormones (estrogen and progesterone) in maternal tissue for embryonic receptivity and tissue regeneration during early gestation period are well established (1). The peri-implantation period in mice is considered critical for successful pregnancy during which embryonic cellular proliferation and differentiation takes place; at the same time change of maternal tissue occurs to accommodate the growing embryo. The changed cellular pattern of embryo and maternal tissue is steered by altered biochemical milieu and physiological processes in precisely coordinated manner between fetal and maternal tissues. In the process, growth factors are expressed by the both tissues in sequential pattern, especially during peri-implantation in mice. Our earlier studies showed that insulin like growth factor (2), transforming growth factor-β (3) and vascular endothelial growth factor (VEGF)-C (4) were expressed in embryo and uterine tissue of albino rat during the peri-implantation period. VEGF is a potent mitogenic factor for endothelial cells and it is expressed in spatial-temporal manner associated with physiological events of angiogenesis (5).

During mouse embryo development, vascular permeability of the uterus facilitates the encounter mechanism of trophectoderm to the uterine luminal epithelium and it is considered as one of the prime pre-requisite for successful implantation process. It is well established that VEGF is associated with uterine vascular permeability, which is sine qua non for attachment of embryonic trophectoderm to the maternal endometrial epithelium, leading to successful pregnancy (6-8). VEGF and the corresponding receptor (VEGFR) actively participate in the process of vascular permeability and decidualization (9), uterine and embryonic angiogenesis (10). VEGF executed its physiological effects, mainly through VEGF receptor-1 known as fms-like tyrosine kinase-1 (Flt-1) and VEGF receptor-2 known as fetal liver kinase-1/kinase insert domain-containing receptor (Flk-1/KDR) (11-14). Research works revealed Flk-1/KDR as a principal receptor of VEGF signal transduction, associated with proliferation and migration of endothelial cells (15-17). Rising permeability at the uterine vasculature and subsequent angiogenesis have vital role in successful implantation, decidualization and placentation (18). 

VEGF is known to be a key candidate in the vascular modification at the uterine bed as well as it has a crucial effect on the survival of embryonic cytotrophoblast in the placenta (19). However, signaling mechanism of the VEGF through VEGFR during mouse embryo development needs to be studied further. In situ localization of VEGF and VEGFR in fetal-maternal tissue of mice during peri-implantation shall provide information on the origin and pattern of cellular proliferation for vasculogenesis during this crucial period of embryonic development. In our earlier study, VEGF-C was shown to be expressed in cell specific manner, in the both embryo and maternal tissuesof mice (20). VEGF-C boundto VEGFR-2 (Flk-1/ KDR), as a criticalrequirement for the movement of embryonic endothelial stem cells from primitive streak to yolk sac: a prerequisite for embryonic blood vessel formation (21).

Research revealed that VEGF and Flk-1/KDR were co-expressed in granulosa cells of ovarian follicles and cultured granulosa cells (22). During follicular and luteal developmental period, differential expression of VEGF was evidenced in granulosa cells and theca cells (23, 24). However, cell specific expression of Flk-1/KDR in uterine as well as fetal-maternal tissues during early gestation period is yet not fully understood. In our present study, in situ localization of VEGFR-2 (Flk-1/KDR) in fetalmaternal tissue of mouse during peri-implantation was studied. The results might provide the insight of cellular localization and sequences of vasculogenesis in mouse embryo, during early gestation.

Extensive research work for the last few decades revealed the crucial role of sex steroids, specially estrogen and progesterone, on successful pregnancy outcome. Investigation of feto-uterine circulation revealed that estrogen has actively participated in the process of angiogenesis and vasodilation, thus regulating the utero-placental blood flow (25). Progesterone also play crucial role in the stromal decidualization; thereby increasing vascular permeability, associated with the uterine vasodilation (26). In the present research work, effects of ovarian steroids on VEGFR-2 expression during this critical period was studied using estradiol-17β antagonist (anti-E2) and progesterone antagonist (anti-P4) administered subcutaneously (S.C.) to female during peri-implantation period. 

Findings of the present research work will help shed light on deciphering the role of VEGF and its receptors in fetal-maternal cross talks as well as embryonic development during early gestation period.

## Materials and Methods

### Animal experiments

The present experimental investigation was done on cyclic female Swiss albino mice (LACA strain). The mice were housed in Departmental Animal Rearing Facility maintaining natural light and dark period. The animals were fed with regular food (Bengal gram, corn flour and vitamin supplement) and water ad libitum and they were treated with antihelmintic drug in every six months. The experimental animal handling protocols were approved by the Institutional Animal Ethical Committee, Rajiv Gandhi University (India) following “Breeding of and Experiments on Animals (Control and Supervision) Rules, 1998”. Mature female mice (25 ± 10 g body weight, 6-8 weeks old), showing normal estrous cycle, were mated with fertile males at the ratio of 2:1 (2 females: 1 male) of the same strain to establish pregnancy. For each group, five mice were used in the present research work (n=5). Pregnancy was confirmed after detection of the vaginal plug and it was considered the first day (D1) of pregnancy upon determining the vaginal plug. Mice were dissected on specific day to collect the uterine samples. Implantation sites on days 4, 5 and 6 were detected by intravenous administration of 0.1 ml 1% Chicago sky blue (SigmaAldrich, USA) in tail vein 15 minutes before necropsy. The implantation sites were demarcated by distinct blue bands. The space between sites of implantation was called inter sites.

### In situ localization of VEGFR-2 (Flk-1/KDR)

During the present research work, immunolocalization of the Flk-1/KDR was performed using HRP-conjugated as well as FITC-conjugated secondary antibody (Santacruz Biotech, USA) following the standard method (27). The immunofluorescence technique was used to corroborate the immunohistochemistry result, since the fluorescence signal was very sensitive and gave precise signal. This was the reason of using both HRP-conjugated and FITC-conjugated secondary antibodies in the present research. Immunohistochemical localization of Flk-1/ KDR was performed in uterine sections embedded in paraffin using HRP and FITC conjugated secondary antibodies. Briefly, the uterine tissues were collected from D4 to D7 of gestations and they were subsequently fixed in bouin’s solution for 72 hours. Following fixation, the tissues were subjected to wash in running tap water for 3-4 hours, dehydrated by passing through different grades of alcohol from 30 to 100% and they were finally kept overnight for paraffin embedding, followed by preparation of blocks. Tissue blocks were sectioned at 5 µm thickness using rotary microtome and they were mounted on glass slides coated with poly-L-Lysine (Sigma-Aldrich, USA). The sections were then kept overnight at 37°C to dry. The sections were deparaffinized for 5 minutes in xylene with two times changing followed by adding isopropanol for 5 minutes. Next, they were rehydrated by passing from different alcohol grades from 100 to 30% and they were thereby washed in Tris-buffered saline (TBS) buffer (10 mM) for 5 minutes with three times changing the buffer. Antigen retrieval was performed by 10 mM citrate buffer (pH=6.0). 

Blocking endogenous peroxidase action was performed by 3% hydrogen peroxide (H2O2) for 30 minutes in methanol. Non-specific binding was prevented by incubating the sections in 10% normal goat serum (NGS, Santacruz Biotech, USA) for 3-4 hours in a humidified chamber at room temperature. Then, the sections were incubated with Flk-1 and mouse monoclonal IgG (both from Santacruz Biotech, USA) primary antibody overnight at 4°C. Primary antibody was diluted in Tris-buffered saline-bovine serum albumin (TBS-BSA) at a concentration of 2.5 µg/ml. The primary antibody treated sections were then washed with TBS buffer for 5 minutes and they were either incubated with HRP-conjugated secondary antibody (Goat antimouse IgG-HRP, Santacruz Biotech, USA) or fluorescein isothiocyanate (FITC) labeled secondary antibody (Goat anti-mouse, IgG-FITC, Santacruz Biotech, USA) for 2 hours. In order to detect signal development for immunohistochemistry, the sections were incubated with diaminobenzidine (DAB) substrate (Amresco, USA) dissolved in TBS and 0.3% H_2_O_2_ at a concentration of 1 mg/ml. Finally, the sections were briefly counterstained with delafield hematoxylin, dehydrated and mounted with D.P.X (Merck, India). 

Similarly, the sections were mounted with an anti-fade mounting media (Santacruz Biotech, USA) for immunofluorescence. Negative control was performed by substituting the primary antibody with normal IgG in the both experiments. The sections were photographed using DM5000B fluorescence microscope (Leica Microsystem, Germany) in different magnifications. Quantitative analysis of the immunofluorescent signals of Flk-1/KDR was studied by using ImageJ software (Version 1.46r, NIH, USA). Intensity values were counted in terms of percentage of the signal. Below 20% values were designated as low intensity (+); 20-40% was considered as moderate (++), 40-80% was defined as high intensity (+++) and above 80% values were considered as intense (++++). The result showed that different cell types of the both embryo and uterine tissues exhibited immunofluorescent signals with varied intensities. 

### Administration of estradiol-17β and progesterone antagonist

Activity of ovarian estrogen was blocked with S.C. injection of estradiol-17β (E2) antagonist ICI 182780 (Sigma-Aldrich, USA) once in a day in between 8 am and 9 am, at a dose of 100 µg/100 µl (28) for three consecutive days from D2 to D4. The treated mice were euthanized on D5 between 8 am and 9 am and uterine tissues were collected. Likewise, progesterone (P4) antagonist RU486 (Mifepriston, Sigma-Aldrich, USA) treated mice received 400 µg/100 µl (28) S.C. between 8 am and 9 am on D4 and D5. Upon euthanasia, the uterine tissues were collected on D6 between 8 am and 9 am, for further studies.

### Western blotting of VEGFR-2 (Flk-1/KDR)

Total uterine proteins were extracted using TRI Reagent (Sigma-Aldrich, USA). The uteri were excised and rinsed with phosphate-buffered saline (PBS) to remove cellular debris. The uteri were homogenized in TRI reagent following the manufacturer’s protocol. Concentration of the protein was detected by Bradford method (Bradford, 1976). The uterine proteins were separated on 12% sodium dodecyl sulphate-polyacrylamide gel electrophoresis (SDSPAGE) using Mini-PROTEAN Tetra cell electrophoresis apparatus (Bio-Rad, USA), following the standard method. 

The separated proteins were transferred onto the nitrocellulose membrane (Optitran BA-S 85, 200 mm×3 mm) using Mini Trans-Blot system (BioRad, USA) at 100 V for 1 hour. Briefly, the membrane was rinsed with Tris-buffered saline and Tween 20 (TBST). Non-specific binding sites were blocked with 5% Blotto non-fat dry milk (Santacruz Biotech, USA) in TBST for 2 hours. The membrane was washed with 1X TBST and incubated in 4°C overnight with the mouse monoclonal Flk-1 (A-3). The membrane was washed with TBST for 5 minutes followed by washing with TBST and TBS (1:1) for 5 minutes with two changes. The membrane was then incubated with HRP-conjugated Goat anti-mouse IgG secondary antibody (Santacruz Biotech, USA) diluted in TBST and TBS (50:50) at room temperature for 2 hours. The membrane was next incubated with tetramethylbenzidine (TMB, Sigma, USA) blotting substrate solution until the band begins to appear. The reaction was stopped by washing the membrane with distilled water. Protein bands were visualized and photographs were taken under ChemiDoc MP System (BioRad, USA). Densitometry analysis of the western blot bands were performed using ImageJ software, using the standard software protocols. 

### RNA isolation and quantitative reverse transcription polymerase chain reaction

Total RNA was isolated from the uterine tissues treated with TRI Reagent, following the manufacturer’s protocol. The isolated RNA was measured in the ratio of 260-280 in NanoDrop 2000 spectrophotometer (Thermo Scientific, USA). RNA samples were reverse transcribed into singlestranded cDNA according to the manufacturer’s instruction using iScript^TM^cDNA synthesis kit (BioRad, USA). Primers were designed using the Primer-3 plus software from National Center for Biotechnology Information database (NCBI; www.ncbi.nlm.nih.gov). The primer sequences for *Flk-1/KDR* include

F: 5'-TTGGAGCATCTCATCTGTTACAGC-3' and

R: 5'-GGCCGGCTCTTTCGCTTACT-3'. 

The primers for *G6PDH* were

F: 5'-TCATGTTTGAGACCTTCAA-3' and

R: 5'-GTCTTTGCGGATGTCCACG-3'.

Real-time quantitative reveres transcription polymerase chain reaction (qRT-PCR) was performed in a 20 µl reaction volume containing 10 µl of 2x iTaq universal SYBR Green supermix (BioRad, USA), 2 µl of each forward and reverse primers, 3 µl DNA template and 3 µl nuclease free water for the quantification of Flk-1/KDR expression using CFX96 Touch Real-time PCR detection system (BioRad, USA). The qRT-PCR program was as following: initial denaturation at 95˚C for 5 seconds, annealing/extension at 60˚C for 30 seconds, as two-steps cycling: 40 cycles; melt curve analysis was performed at 95˚C for 5 seconds and polymerase activation/DNA denaturation at 95˚C for 30 seconds. Finally, data were analyzed using the BioRad CFX Manager software.

### Statistical analysis

Densitometry data of immuno-signal intensity, western blotting and qRT-PCR product of *Flk-1/KDR* were statistically analyzed using the statistical software (SPSS, version 10.0, SPSS Inc. USA). Western blot and qRT-PCR densitometry data on D4 of gestation was compared to the other gestation days. All data were presented as mean value ± standard error (SEM). One-way ANOVA was performed and it was followed by Tukey post hoc test to find the significant difference. All differences were considered statistically significant, providin g P<0.05.

## Results

### In situ localization of VEGFR-2 (Flk-1/KDR)

### Day 4 (D4)

**Fig.1:**
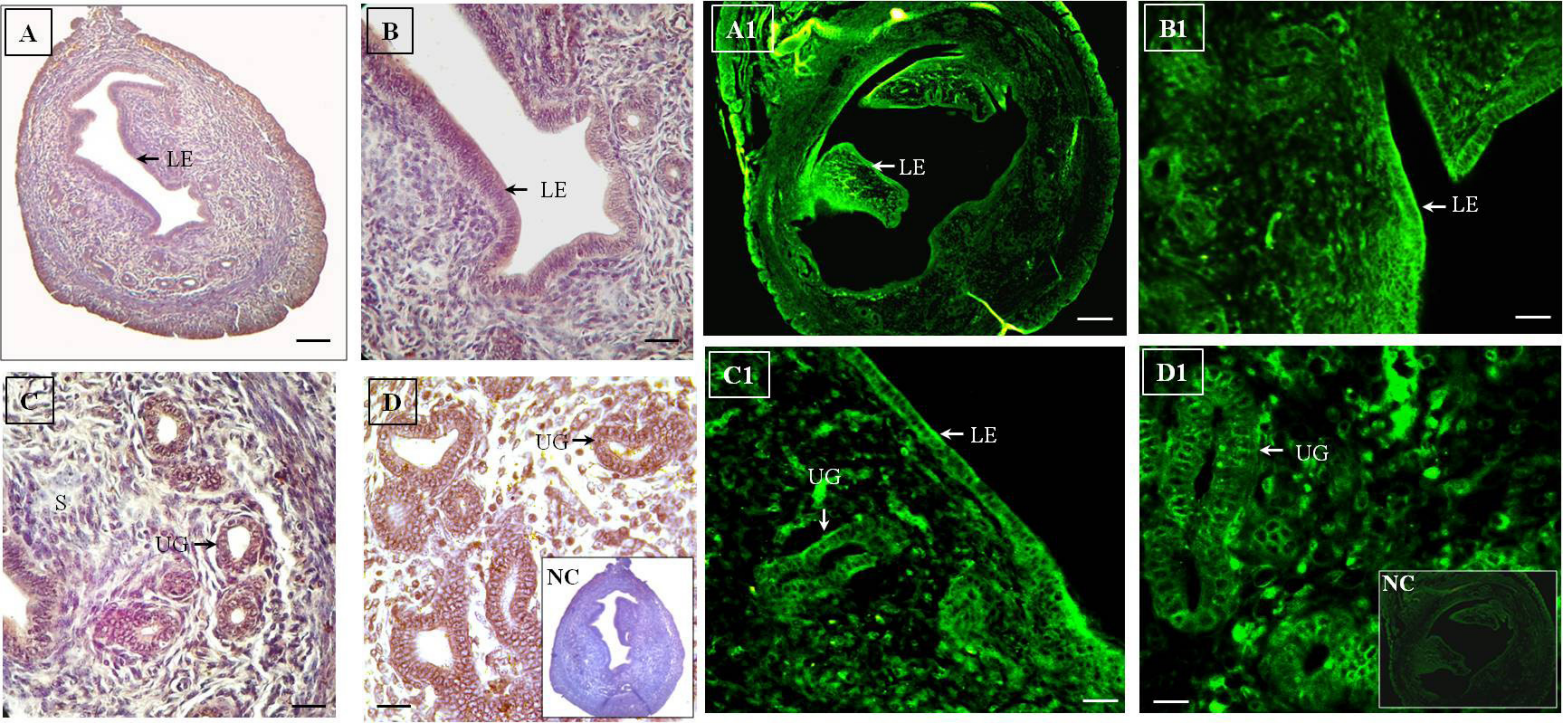
Immunohistochemistry and immunofluorescence of D4 mice uterus. **A-D.** Immunohistochemical localization of Flk-1/KDR on D4 mice uterus using HRP-conjugated antibody. The receptor is localized in the LE, UGs and S. The expression is more in LE and glandular epithelium compared to stromal cells. **A1-D1.** These figures depicts immunofluorescence of Flk-1/KDR on D4 mice uterus using FITC-conjugated antibody. The receptor is localized in LE, UGs and S. The expression pattern is similar to the HRP-conjugated antibody. LE and glandular epithelium exhibit strong expressions of the receptor (original magnification: A, A1: 5x, B, C, B1, C1: 20x, D, D1: 40x, scale bar: A, A1: 100 μm, B, C, B1, C1: 50 μm, D, D1: 10 μm). LE; Luminal epithelium, UG; Uterine glands, S; Stroma, and NC; Negative control.

The uterine tissue treating with HRP-conjugated antibody on D4 of gestation (Fig.1A-D) showed strong expression of Flk-1/KDR in the luminal and uterine glandular epithelium. Stromal cells expressed the receptor with moderate intensity. Myometrium and perimetrium also exhibited immunostaining of Flk-1/KDR on D4 of gestation. From D4 of gestation onwards, the stromal cells underwent dramatic morphological and biochemical changes which were considered as prerequisite for implantation of the growing embryo to the maternal tissue.

Immunofluorescence of Flk-1/KDR using FITC-conjugated secondary antibody is presented in the Figure 1A1- D1. The result showed proliferative endometrial epithelium with distinct epithelial cell layers. The immunofluorescence result revealed presence of the receptor in the luminal epithelium, uterine glands and stroma similar to that of the HRP-conjugated antibody. Stromal cells in close vicinity of the endometrial epithelium exhibited higher intensity of the Flk-1/KDR expression. It was observed that expression of the receptor was higher in the stromal cells surrounding the uterine glands compared to that of the other areas. Expression of FITC-conjugated antibody was corroborative with that of the HRP-conjugated antibody in the uterine tissue sections on D4 of gestation. Signal analysis revealed that expression of Flk-1/KDR was strong in the luminal epithelium and uterine glands (Table S1, See Supplementary online Information at www.ijfs.ir). 

**Fig.2:**
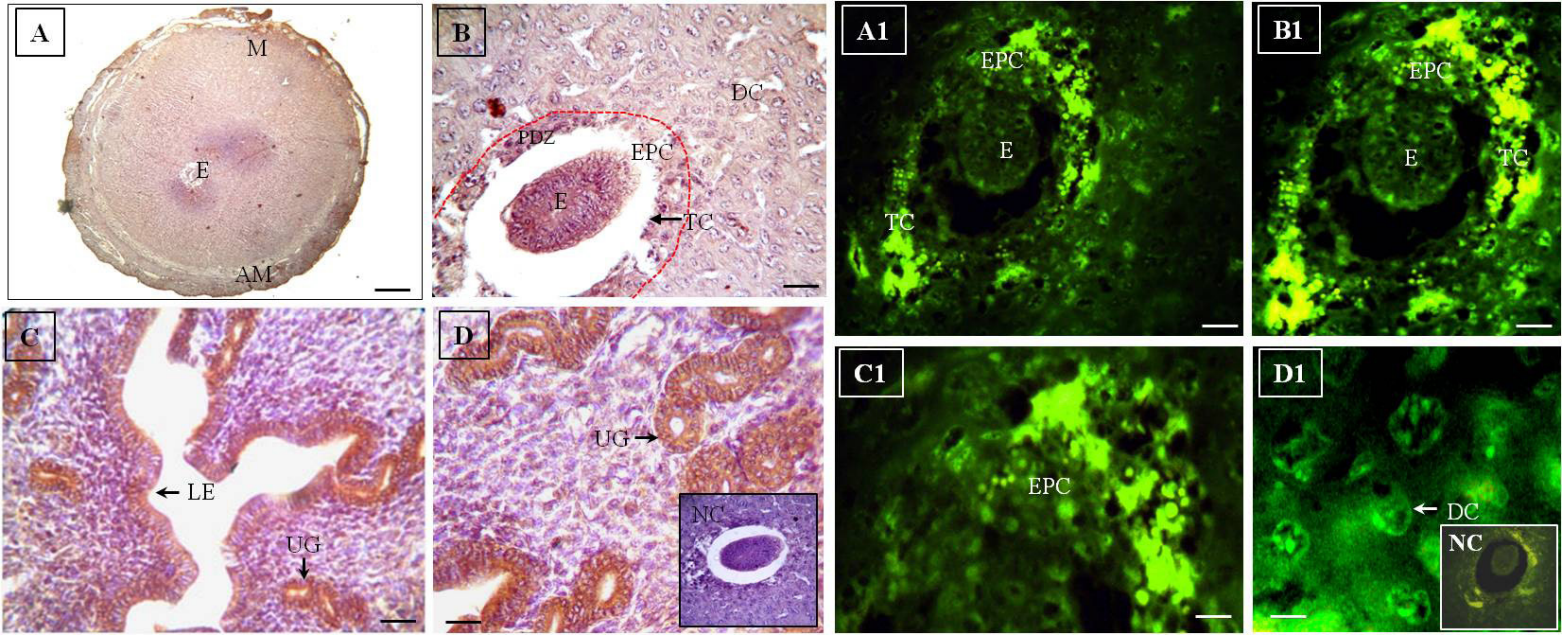
Imunohistochemistry and immunofluorescence of D5 mice uterus. **A-D.** Immunohistochemistry of Flk-1/KDR on D5 of gestation using HRP-conjugated antibody. LE, UGs and Sexpress Flk-1/KDR. E and TC also express the receptor. LE and UGs on D5 express more Flk-1/KDR in comparison with D4. However, expression of the receptor is more intense in the glandular epithelium than LE on D5 of gestation. The red dotted line showed PDZ. **A1-D1.** These figures showed immunolocalization of Flk-1/KDR on D5 of gestation, using FITC conjugated secondary antibody. The receptor is localized strongly in the TC and the DC surrounding the embryo. The emerging EPC also express Flk-1/KDR. The embryonic cells exhibit expression of the growth factor receptor (original magnification: A: 5x, B, C: 20x, D, C1, D1: 40x, A1, B1: 10x, scale bar: A: 100 μm, B, C, A1, B1: 50 μm, D, C1, D1: 10 μm). M; Mesometrium, E; Embryo, AM; Anti-mesometrium, LE; Luminal epithelium, UG; Uterine glands, PDZ; Primary decidual zone, EPC; Ectoplacental cone, DC; Decidual cell, TC; Trophectodermal cell, and NC; Negative control.

### Day 5 (D5)

After successful implantation of the blastocyst to the uterine endometrial epithelium on D4.5, the embryo appeared as an oval structure on D5 of gestation (Fig.2). It was observed that embryo attached to the mesometrial pole in the maternal epithelium and gradually invaded to the maternal tissue. The luminal epithelium appeared to be apposition with each other. The decidual cell (DC) reaction was started during this period and the stromal cells were gradually changed to round structure and transformed to DC. The decidualization process was firstly started at the stromal cells surrounded the embryo forming primary decidual zone (PDZ). The trophectodermal cells were attached to the maternal wall creating a fluid filled blastocyst cavity (BC) for accommodation of the embryo. The inner cell mass of blastocyst gradually proliferate, forming embryo in the BC surrounded by trophoblast cells (TCs). During this period, ectoplacental cone (EPC) formation was started at the mesometrial site.

Using HRP-conjugated secondary antibody, immunohistochemistry showed distinctive expression of the receptor in different embryonic cell types, maternal tissues and fetal-maternal interface (Fig.2A-D). The receptor was localized in the embryonic trophectodermal cells as well as developing EPC with moderate intensity on D5 of gestation (Fig.2A, B). The cells surrounding the implanted embryo occupied a distinctive region of DCs forming PDZ, as mentioned earlier. DCs surrounding embryo showed strong Flk-1/KDR immunostaining (Table S1, See Supplementary online Information at www.ijfs.ir). It was also demonstrated that DCs surrounding the embryonic pole of embryo exhibited strong immunostaining of Flk-1/KDR, compared to that of the anti-mesometrial side. Interestingly, the glandular epithelium exhibited intense immunostaining of Flk-1/KDR, compared to that of the luminal epithelium (Fig.2C, D).

Cell specific immunolocalization of Flk-1/KDR in the fetal-maternal tissues and embryo on D5 of gestation was established by the immunofluorescence study, using FITC-conjugated secondary antibody (Fig.2 A1-D1). Immunofluorescence study revealed strong expression of the receptor at the developing EPC. DCs of PDZ showed strong expression of the receptor. Trophectodermal cells surrounding embryo showed strong expression of Flk-1/ KDR. Moreover, both the mural and polar TCs of embryo exhibited significant expression of Flk-1/KDR on D5 of gestation. It was also observed that the embryonic cells expressed the Flk-1/KDR receptor with moderate intensity (Table S1, See Supplementary online Information at www.ijfs.ir).

### Day 6 (D6)

**Fig.3:**
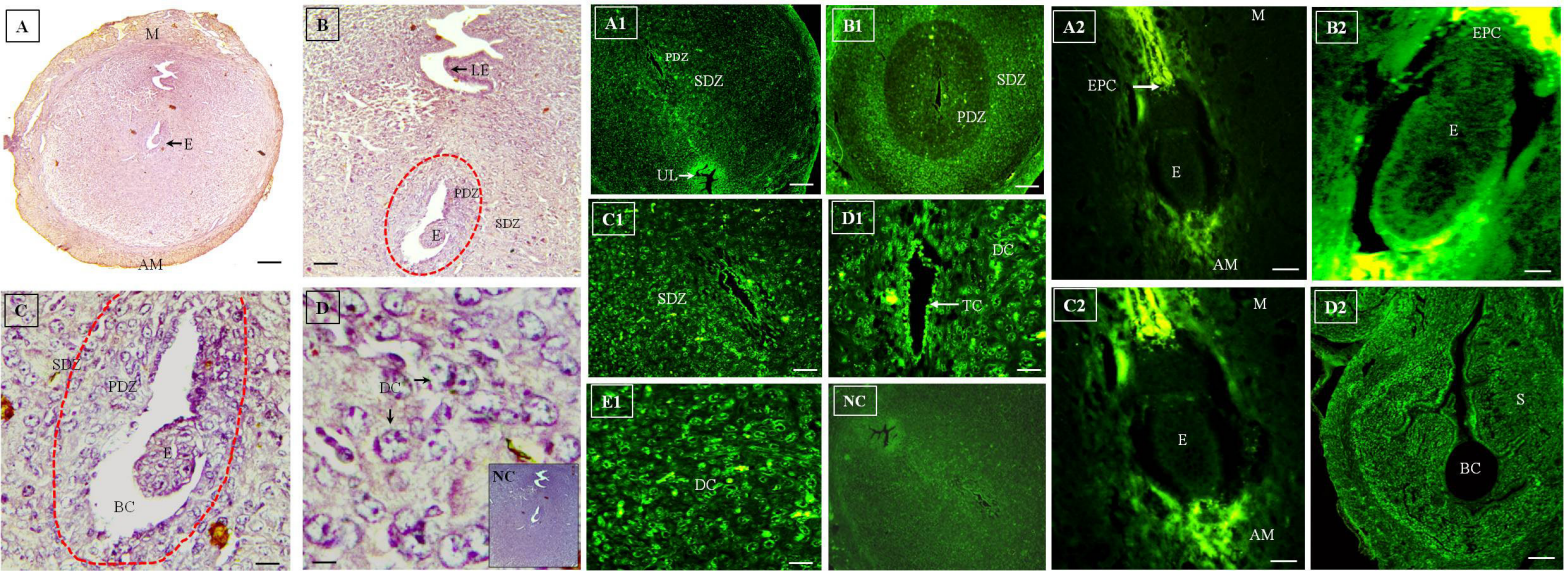
Immunohistochemistry and immunofluorescence of D6 mice uterus. **A-D. **Immunohistochemical localization of Flk-1/KDR on D6 mice uterus and fetal maternal tissues using HRP-conjugated antibody. The receptor is localized in PDZ and SDZ. LE and E cells, especially the cells bordering the embryo exhibit more immunostaining of Flk-1. DC also exhibit expression of the receptor. **A1-E1.** These figures depicts immunolocalization of Flk-1/KDR on D6 of gestation using FITC conjugated antibody. DCs of the SDZ express more Flk-1/KDR compared to PDZ. The receptor is localized in TC and DC surrounding the embryo. E cells exhibit expression of the receptor. **A2-D2.** These figures showed immunofluorescence of Flk-1/KDR on D6 of embryo using FITC conjugated antibody. The receptor is localized in the M and AM sides surrounding the embryo. E cells also express Flk-1/KDR. Intensity of the expression is strong in LE. EPC also express Flk-1/KDR with high intensity. BC is formed to accommodate the developing embryo (original magnification A, A1, B1, D2: 5x, B, C1, A2: 10x, C, C2: 20x, D, D1, E1, B2: 40x, scale bar: A, A1, B1: 100 μm, B, C, C1, A2: 50 μm, D, D2: 100 μm, D1, E1, B2, C2: 10 μm). PDZ; Primary decidual zone, SDZ; Secondary decidual zone, LE; Luminal epithelium, E; Embryo, DC; Decidual cell, M; Mesometrial, AM; Anti-mesometrial, EPC; Ectoplacental cone, BC; Blastocyst cavity, UL; Uterine lumen, TC; Trophoblast cell, S; Stroma, and NC; Negative control.

A rapid morphological as well as spatial change of the stromal cells occurred with the progression of post-implantation gestation days. On D6 of gestation, the area of DCs was expanded from PDZ towards the myometrium. This expanded decidual zone is called secondary decidual zone (SDZ). The embryo attached to the maternal wall through trophoblastic cells of EPC. During this period of gestation, the luminal epithelium within implantation site was degraded, due to the invasion of the trophoblasts resulted in tearing of the maternal stroma. The expression was detected by HRP-conjugated antibody in PDZ cells of the implantation sites (Fig.3). The trophectodermal cells surrounding BC and the embryo cells, especially those bordering the embryo, exhibited strong immunostaining of Flk-1/KDR receptor. Higher expression of Flk-1/KDR at the decidual zone associated with differentiation and migration of DCs to form SDZ. It was observed that expression of the receptor was more in the PDZ. Moreover, the expression was low in SDZ, compared to PDZ. Trophectodermal cells and certain cells of the embryo exhibited significant receptor expression. Maternal luminal epithelium exhibited strong expression of the Flk-1/KDR. 

Study of Flk-1/KDR (using FITC-conjugated antibody) on D6 of gestation showed that the receptor was expressed in the cells of fetal-maternal interface (Fig.3 A1-E1). It was observed that embryonic cells bordering embryo exhibited significant expression of the Flk-1/KDR. During this period, PDZ showed higher expression of receptor, in comparison with SDZ. Trophectodermal cells of the embryo also exhibited expression of the Flk-1/KDR. TCs of mesometrial and anti-mesometrial side surrounding the embryo exhibited strong expression of the receptor (Fig.3 A2-D2).

**Fig.4:**
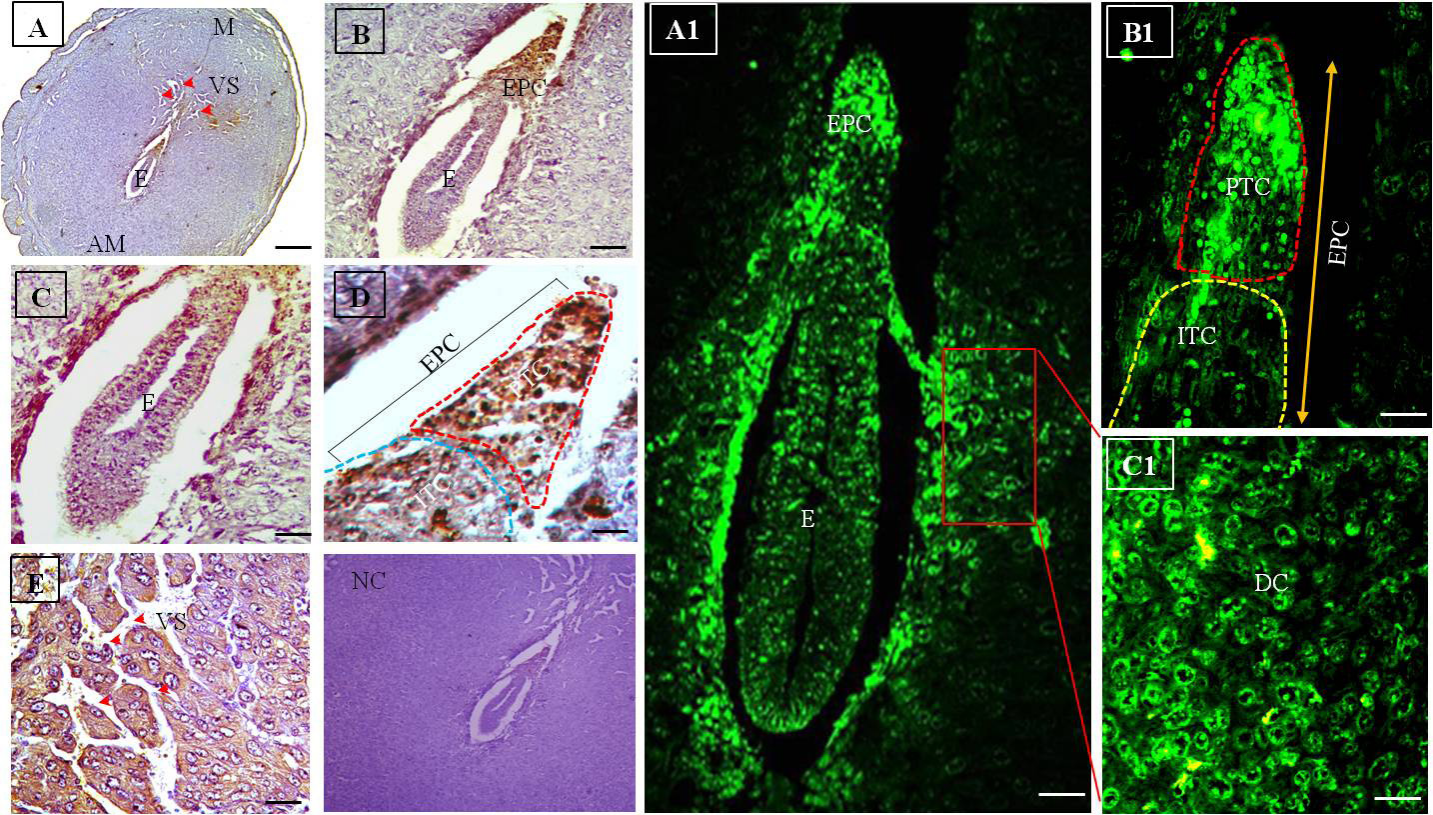
Immunohistochemistry and immunofluorescence of D7 mice uterus. **A-E.** Immunohistochemical localization of Flk-1/KDR on D7 mice uterus and fetal maternal tissues using HRP-conjugated antibody. The receptor is localized in the E cells, specifically in EPC. E cells towards EPC exhibit more immunostaining than the embryonic cells of the anti-mesometrial side. Expression of the receptor in the cell layering VS of M side revealed possible involvement of the receptor in placenta development. Arrow heads show presence of receptor towards M side. The red dotted line depicts the PTCs and blue dotted line depicts the ITCs of the EPC. **A1-C1.** These figures depicts immunohistochemical localization of Flk-1/KDR on D7 mice uterus and fetal maternal tissues using FITC-conjugated antibody. The receptor is localized in the cells of the embryo and specifically in EPC. E cells towards the EPC exhibit more immunostaining than the embryonic cells of anti-mesometrial side. The red dotted line depicts PTCs and yellow dotted line depicts ITCs (original magnification: A: 5x, B: 10x, C: 20x, D, E, A1-C1: 40x, scale bar: A: 100 μm, B, C: 50 μm, D, E, A1-C1: 10 μm). E; Embryo, EPC; Ectoplacental cone, VS; Vascular sinusoids, M; Mesometrium, PTC; Peripheral trophoblast cell, ITC; Inner trophoblast cells, AM; Anti-mesometrium, DC; Decidual cell, and NC; Negative control

### Day 7 (D7)

On D7 of gestation, there was a drastic morphological change of the embryo as well as the maternal tissues. During this period, embryo was elongated in shape with proper development of EPC in the mesometrial side (Fig.4). During D7 of gestation, EPC was rapidly developed and it was morphologically organized in distinct cell populations: the inner and peripheral cells, as shown in Figure 4D and B1. During D7, VS were extensively formed at the mesometrial side. Strong immunostaining of the Flk1/KDR in the VS at the mesometrial side was observed. It is believed that sinusoids provide surface area for the developing embryo. Immunohistochemical localization of Flk-1/KDR, using HRP-conjugated antibody, showed that the receptor was specifically confined in the EPC with high intensity. In this stage, PDZ was gradually degraded and overlaid by SDZ. Peripheral TCs (PTCs) of the EPC expressed more Flk-1/KDR immunostaining, in comparison with the other area. It was determined that different cell types of the embryo also expressed the receptor with diverse intensities. Different cell types of EPC expressed Flk-1/KDR with diverse intensities. PTCs of the EPC expressed strong immuno-signal of Flk-1/KDR, compared to the inner TCs (ITCs) of EPC (Fig.4B1). Moreover, the TCs bordering EPC exhibited strong expression of receptor. At this stage, the embryo was elongated in the specific orientation. Embryonic cells were differentiated into three germinal layers with formation of inner cleft. Different cell types of the embryo also exhibited various intensities of the Flk-1/KDR expression. DCs surrounded the developing embryo also expressed the receptor. 

### Expression of VEGFR-2 (Flk-1/KDR) following antiE2 and anti-P4 administrations

**Fig.5:**
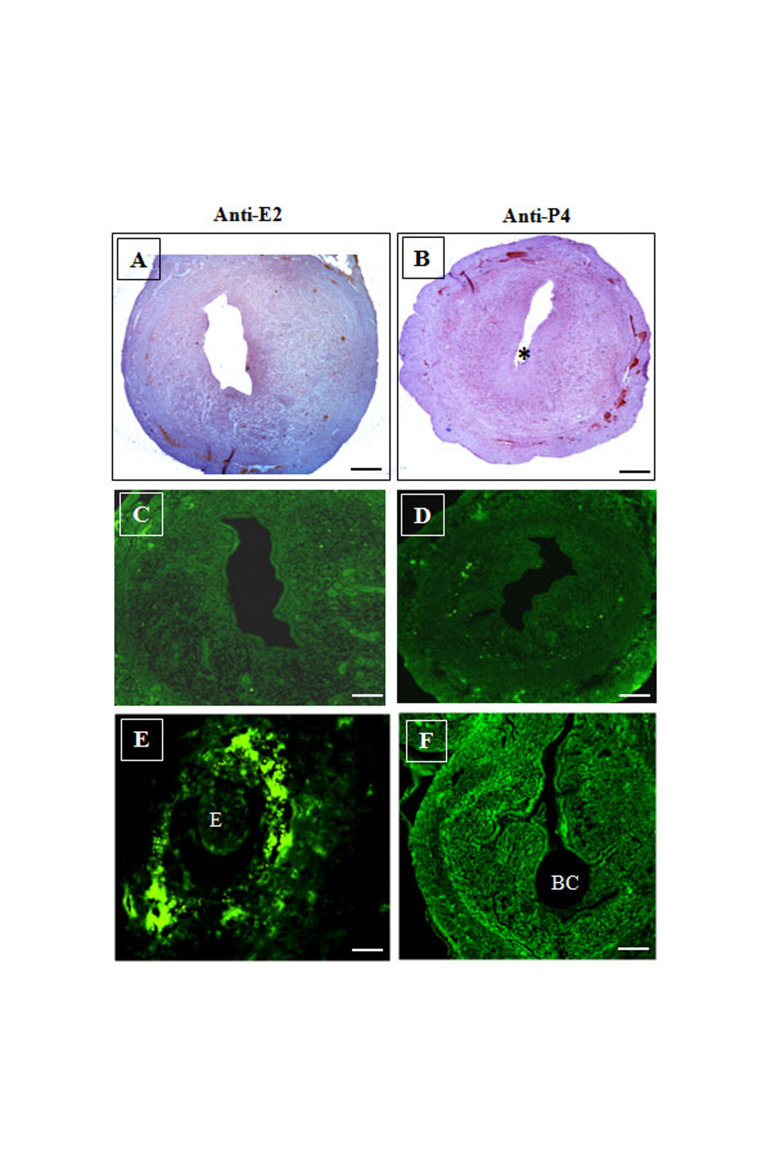
Immunohistochemistry and immunofluorescence of anti-E2 and anti-P4 treated mice uterus. **A.** Immunohistochemistry of Flk-1/KDR using HRP-conjugated and FITC-conjugated antibody on anti-E2 treated (**A** and **C**) and anti-P4 treated (**B** and **D**) mice uterus. The figure showed that Flk1/KDR was not expressed in the anti-E2 and anti-P4 treated uterus. **E, F.** These figure showed normal pregnant mice with five and six days embryo and BC for comparing with E2 and P4 antagonist treatment. Asterisk (*) showed underdeveloped BC (scale bar: 50 μm). E2; Estrogen, P4; Progesterone, E; Embryo, and BC; Blastocyst cavity.

Expression of Flk-1/KDR in the uteri of females administered with anti-E2 and anti-P4 is presented in Figure 5. Anti-E2 treatment was given from D2 to D4 and uterine samples were collected on D5. As expected, findings showed no sign of implantation, due to the antagonistic blocking of the E2 receptor in the uterine cells. No immuno-signal of Flk-1/KDR was observed in the antiE2 treated uterine sections (Fig.5A, C). Anti-P4 injection was administered on D4 and D5, followed by sacrificing the female mice on D6 of gestation. Due to the blocking P4 receptor, there was failure of implantation. Thus, the pregnancy developmental process could not proceed and the embryo was failed to develop. Therefore, anti-P4 treated D6 uterus was determined under developed BC without presence of the embryo, as shown in Figure 5B. The implanted blastocyst could not survive, due to the absence of P4 signaling activity. In this research, Flk-1/ KDR expression was not detected in the anti-P4 treated mice uterus (Fig.5B, D).

### Western blotting of VEGFR-2 (Flk-1/KDR) 

**Fig.6:**
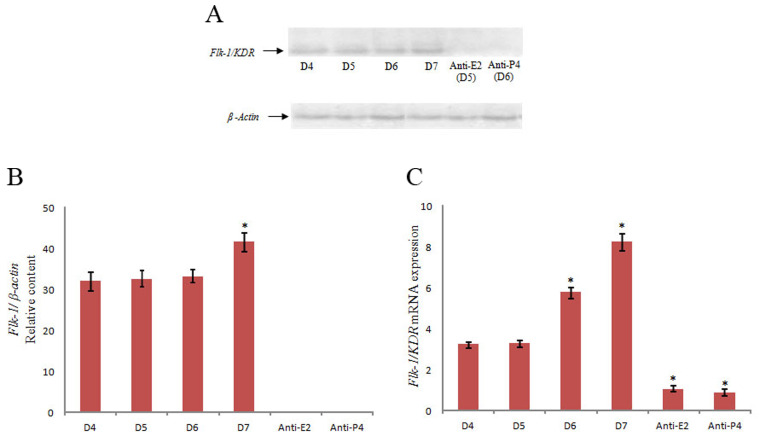
Western blotting and qRT-PCR analysis of Flk-1/KDR. **A.** Western blotting of Flk-1/KDR during peri-implantation period (D4-D7) of gestation and in E2 and P4 antagonist treated uterus. **B.** Densitometric analysis of western blotting result of Flk-1/KDR protein. The result showed significant increase of the receptor on D7 when compared to D4. All experiments are repeated three times. Data are shown as mean ± SEM (P<0.05), compared to D4. **C.** qRT-PCR of *Flk-1/KDR* during D4-D7 of gestation, in addition to the E2 and P4 antagonist treated uterus. The result revealed that *Flk-1*mRNA level was up-regulated with progression of gestation days. There are significant upregulations of *Flk-1* mRNA on D6 and D7, compared to D4. The result also revealed significant down-regulation of Flk-1 mRNA transcript after treatment of E2 and P4 antagonist, suggesting that Flk-1/KDR expression during periimplantation was estrogen and progesterone dependent. All of the experiments are repeated three times. Data are shown as mean ± SEM (P<0.05) compared to D4. E2; Estrogen, P4; Progesterone, and qRT-PCR; Quantitative reverse transcriptase- polymerase chain reaction.

Western blot analysis showed Flk-1/KDR protein in the all gestation days (D4-D7) of the experimental period. It was found that intensity of protein bands was gradually increased in western blot from D4 to D7, demonstrating higher expression of the receptor with progression of gestation days (Fig.6A, B). β-actin was used as loading control during this experiment. The results showed that Flk-1/ KDR reached to the maximum level on D7 of gestation, in comparison with D4. Densitometric analysis showed that expression level of Flk-1/KDR on D7 was significantly higher than D4 (P<0.05) of gestation. No protein expression was detected for the anti-E2 and anti-P4 uterus

### Quantitative reverse transcription polymerase chain reaction of *VEGFR-2* (*Flk-1/KDR*)

Transcription pattern of *Flk-1/KDR* mRNA in the mice uteri during peri-implantation period (D4-D7) was studied by qRT-PCR using both forward and reverse gene specific primers. The results exhibited increasing pattern of the mRNA transcript during the period of peri-implantation (Fig.6C). qRT-PCR data showed that *Flk-1/KDR* mRNA transcript was up-regulated with progression of the gestation days. There were significant up-regulations of Flk-1 mRNA on D6 and D7, compared to D4 (P<0.05). *Flk-1/KDR* mRNA transcript was up-regulated almost four times on D7, compared to D4. Similarly on D6, *Flk1/KDR* mRNA transcript was up-regulated almost three fold, compared to D4 of gestation. The result also revealed significant down-regulation of *Flk-1* mRNA transcript after treatment of E2 and P4 antagonist, suggesting that *Flk1/KDR* expression is estrogen and progesterone dependent during peri-implantation. All of the experiments were repeated three times. Data are depicted as mean ± SEM (P<0.05), compared to D4. 

## Discussion

In the present study, VEGFR-2 (Flk-1/KDR) signal was detected in the cell specific manner from D4 gestation onward. In rodents, blastocyst implantation takes place on D4.5 of gestation. This period is characterized by high degree of uterine receptivity along with stromal cell proliferation and its gradual conversion to DC. Estrogen, progesterone and certain growth factors are decisively involved in causing receptive uterus for successful blastocyst implantation on D4.5 (29, 30). Expression of VEGFR-2 (Flk-1/KDR) in uterine epithelium prior to implantation suggests involvement of VEGF in window preparation, neo-angiogenesis and recruitment of leucocytes as well as invasion of the trophoblasts. Subsequently, after implantation, significant proliferation and differentiation of the stromal cells occur, culminated in surrounding embryo with large, polyploid nucleus. During this period, vascular dilation occurs at the endometrial epithelium which facilitates invasion of the blastocyst to the maternal stroma. This decisive event is regulated by VEGF signaling through VEGFR-2 (Flk-1/ KDR) (5, 31). 

VEGFR-2 expressed on the embryonic trophectodermal cells as well as the maternal tissue at mesometrial and anti-mesometrial pole. The embryonic trophoblastic cells at the mesometrial end and in the fetal-maternal interface gradually transformed to trophoblastic giant cells, which gradually invaded to the maternal tissues. The invasion of TCs was accompanied by maternal tissue apoptosis. TCs lining BC which are also called mural TCs (MTC) and possess the apoptotic ability. This invasion mechanism of the trophoblastic cells establishes a fetal-maternal vascular relationship which facilitates proper nutritional support of developing embryo (1). During this period, due to the invasion of the embryo, vascular sinusoids (VS) starts to appear at the mesometrial side, leading to surface area increase for further invasion of the developing embryo to the maternal tissues. TCs towards the embryonic pole are also called polar TC, gradually started to develop EPC. An outgrowth of TC formed top of the embryonic pole capping the embryoblast. These TCs are cuboidal in shape and form a cap-like outgrowth which ultimately give rise to EPC. Peripheral trophoblastic cells exert an apoptotic effect and eventually break up the adjoining uterine epithelium. Degeneration of the stromal cells are extended all around the egg cylinder that leads to the proliferation of polar TCs and ultimately forming EPC. During this period, VEGFR-2 (Flk-1/KDR) is expressed in an ordered manner, in both embryonic and maternal tissues. Recent study revealed that VEGF plays crucial roles in BC formation, development of blastocyst cells and growth during early gestation in mice (32). It is believed that the ligand VEGF does the physiological events signaling through VEGFR-2 for programmed growth and development of the embryo. Our earlier studies showed similar spatial-temporal expression of VEGF-C in fetalmaternal tissue during this crucial episode of gestation (20). It is believed that VEGF and VEGFR-2 plays pivotal role in survival of the embryo during this critical period of embryonic invasion and growth. In our earlier study, it was showed that any agent inducing change in biochemical milieu of maternal tissue could lead to embryonic death and loss of normal VEGF expression. Moreover, oral administration of methanolic crude bark extract of *Dysoxylumalliarium* to female rat from gestation D1 led to embryonic death and altered expression of VEGF in both fetus and maternal tissues (4).

From D6 of gestation onward, DCs started to become multinucleated. The multinucleated DC facilitated high degree of protein synthesis which was a prime requirement for the nourishment of the developing embryo (1). The TCs of mesometrial and anti-mesometrial side neighboring the embryo exhibited strong expression of Flk-1/KDR. Mesometrial TCs associated with gradual invasion of developing embryo towards the mesometrial side, forming VS and became proliferated resulting in formation of placenta. Anti-mesometrial TCs gave rise to form primitive placenta in mice and rodents (21). Moreover, decidualization of uterine stromal cells in mice underwent specialized processing that resulted in the formation of giant multinucleated cells by repeated DNA replication, but without cell division called endoreduplication. This process required transition from mitotic cycle to the endoreduplication cycle (1, 33). Our present study showed that Flk-1/KDR expression in the multinucleated DCs on D6 of gestation. The multinucleated DCs gradually underwent degeneration, especially at anti-mesometrial end causing remodeling of the implantation chamber (34). This degeneration facilitated TCs to access maternal blood vessels. In the present investigation, accumulation in the giant DCs suggested VEGFR-2 protein role in establishment of fetal-maternal cellular relationship through vasculogenesis for further development. It is also evident that expression of VEGFR-2 is high in the mesometrial and anti-mesometrial side neighboring the embryo on gestation D6. Moreover, earlier research work showed that on D6, extensive proliferation of the stromal cells exterior to the PDZ occured to appear SDZ around the PDZ. This specialized zone was fully developed in D7 and PDZ was gradually degenerated by apoptosis (35).

Polar TCs of embryo are highly proliferative in nature. Proliferation of the polar TCs subsequently gives rise to a cap like outgrowth that protrudes into the mesometrial side and ultimately forms EPC. Formation of EPC is the first sign of placental development. During D7 of gestation, EPC rapidly developed and morphologically organized in distinct cell populations: the ITCs and the PTCs. ITC of EPC exhibits intense proliferative activity facing the ectoplacental cavity while in the outer regions, PTCs arises. These TCs are non-proliferating, polyploid, invasive and phagocytic in nature. Our present study showed that PTCs of EPC expressed Flk-1/KDR with strong intensity. Specific expression of VEGFR-2 (Flk1/KDR) in the EPC suggested therole of this protein in differentiation and transformation of cells during placental formation. Research work revealed that Flk-1 null mice died between D8.5 and D9.5, due to inability of developing proper blood vessel network (36). In the present study, Flk-1/KDR expression in various cells of the both fetal and maternal tissues suggested the role of this receptor in establishment of fetal-maternal communication during early gestation period. 

It is well established that implantation is a steroid hormone dependent process. This hormone action is essential for preparation of endometrium, among the successful implantation. Proper timing of implantation is very essential for establishment of pregnancy and receptivity of the uterus is attributed by estrogen and progesterone. It was found that estrogen could initiate implantation process. Estrogen is the critical determinant of the implantation process as estrogen primes on D4uterus triggers the uterine receptivity. Additionally, duration of the implantation window depends on the concentration of estrogen. Lower estrogen levels extend the implantation window, while higher level causes rapid close of the window (37). In our experiment, it was found that receptor is not localized in the anti-E2 and anti-P4 treated uterus. This finding suggested that Flk-1/ KDR expression in the uterus, during peri-implantation was gonadal steroid dependent. Western blotting result revealed presence of the receptor in the all gestation days. qRT-PCR study showed significant up-regulation of the receptor with progression of the gestation days. Significant down-regulation of *Flk-1/KDR* mRNA in the estrogen and progesterone antagonist treated uterus, further proved that VEGFR-2 expression in the early pregnancy period was gonadal steroid dependent. 

## Conclusion

Using the findings obtained from the present research work, we can concluded that spatial-temporal expression of Flk-1/KDR during peri-implntation period in mice uterus, especially in the feto-maternal interface, played a critical role in the blastocyst implantation as well as the embryonic development which led to the successful pregnancy. This spatio-temporal specificity could be one of the determinants for establishment of fetal-maternal cross talk during the critical period of development.

## Supplementary PDF


