# Supplementary material for: Cell Specific Expression of Vascular Endothelial Growth Factor Receptor-2 (Flk-1/KDR) in Developing Mice Embryo and Supporting Maternal Uterine Tissue during Early Gestation (D4-D7)
Source: Int J Fertil Steril. 2021 Mar 11;15(2):148–57. doi: 10.22074/IJFS.2021.134530 (PMC8052796; doi:10.22074/IJFS.2021.134530)
Supplement: Supplementary file 1 [file Int-J-Fertil-Steril-15-148-s01.pdf]

Int J Fertil Steril
International Journal of Fertility & Sterility
ISSN:2008-076X eISSN:2008-0778

Supplementary Information for

Cell Specific Expression of Vascular Endothelial Growth Factor Receptor-2 (Flk-1/KDR) in Developing Mice Embryo and Supporting Maternal Uterine Tissue during Early Gestation (D4-D7)

Dimpimoni Das, M.Sc.[1], Purba J Saikia, Ph.D.[2*], Upasa Gowala, M.Sc.[1], Hirendra N Sarma, Ph.D.[1]

1. Molecular Endocrinology and Reproductive Biology Research Laboratory, Department of Zoology, Rajiv Gandhi University, Itanagar, Arunachal Pradesh, India
2. Department of Zoology, Dhemaji College, Dhemaji, Assam, India

Table S1: Quantitative analysis of immunohistochemistry signals of Flk-1/KDR in cell types of embryo and maternal tissue during D4-D7 of gestation. Intensity values are expressed as means of observations (n=10)

	Embryo	Stroma	Luminal Epithelium	PDZ	SDZ	Ectoplacental Cone	Endometrial Gland
D4	----	++	+++	----	----	----	+++
D5	++	++	+++	+++	----	++	++++
D6	++	++	+++	+++	++	+++	----
D7	+++	----	----	+++	+++	++++	----
Anti-E2	----	----	----	----	----	----	----
Anti-P4	----	----	-----	----	----	----	----

+ < 20% (Low) ; ++ 20 % - 40% (moderate) ; +++ 40 % - 80% (Strong) ; ++++ > 80% (Intense)

Received: 25 October 2019, Accepted: 26 September 2020
*Corresponding Address: Department of Zoology, Dhemaji College, Dhemaji, Assam, India
Email: purbajyoti81@gmail.com
